# Mechanical Feasibility Study of Pressed and Burned Red Ceramic Blocks as Structural and Sealing Masonry

**DOI:** 10.3390/ma15145004

**Published:** 2022-07-19

**Authors:** Niander Aguiar Cerqueira, Victor Souza, Jonas Alexandre, Gustavo de Castro Xavier, Roman Fediuk, Sergio Neves Monteiro, Marcelo Neves Barreto, Afonso R. G. de Azevedo

**Affiliations:** 1 FAETEC—Technical School Support Foundation, Av. 28 de Março, Campos dos Goytacazes 28020-740, Brazil; coord.niander@gmail.com; 2 Department of Mechanical Engineering, School of Engineering, Praia Vermelha Campus, UFF-Federal Fluminense University, Rua Passos da Pátria, Niterói 24210-240, Brazil; victor_souza11@hotmail.com; 3 LECIV-Civil Engineering Laboratory, UENF-State University of the Northern Rio de Janeiro, Av. Alberto Lamego, 2000, Campos dos Goytacazes 28013-602, Brazil; jonasuenf@gmail.com (J.A.); gxavier@uenf.br (G.d.C.X.); 4 Polytechnic Institute, Far Eastern Federal University, 690922 Vladivostok, Russia; roman44@yandex.ru; 5 Peter the Great St. Petersburg Polytechnic University, 195251 St. Petersburg, Russia; 6 IME—Materials Science Program, Military Engineering Institute, Praça Gen. Tibúrcio, 80, Urca 22290-270, Brazil; snevesmonteiro@gmail.com; 7 IFF–Fluminense Federal Institute, Campus Centro, Parque Tamandare, Campos dos Goytacazes 22150-140, Brazil; marceloneves2507@gmail.com

**Keywords:** PBB, structural masonry, quality parameters

## Abstract

In the search for better constructive efficiency and a reduction of the waste of construction materials, several researches have been performed in the last years around the world. Red ceramic blocks are artifacts widely used in civil construction around the world, and they result in a great consumption of raw materials and energy. The great innovation of this research was the development of ceramic blocks through an innovative method of pressing and dosing materials, replacing the traditional stage of extrusion in the manufacture of ceramics. In such a sense, a new manufacturing technology for ceramic blocks was proposed through the pressing process, adapting the soil-cement brick press machine, thus attaining more even pieces with greater compliance to the dimensions and preset geometry. In this work, the physical and mechanical features of the pressed and burned blocks (PBB) are produced in a partnership with *Arte Cerâmica Sardinha*, a traditional ceramic industry in the region of Campos dos Goytacazes, RJ, Brazil. It was sought to set the quality parameters for the blocks, to set their mechanical compressive strength, deformation modules and the Poisson coefficient. The blocks were tested in use by means of three layers of prism and small wall samples, and it was checked the fragile-type failure of the PBB. Results indicate that the blocks can be employed in small-sized construction works, as the characteristic compressive strength to block measured was 3.62 N/mm^2^ for average water absorption of 20.84%.

## 1. Introduction

The construction industry has experienced great advances in recent decades, particularly regarding the use of new materials, which allow faster construction processes, lower costs and, at the same time, a high standard of quality, thus preserving efficiency and aiming at profitability and customer satisfaction. In the search for construction systems that may comply with the requirements of a competitive and demanding market, it is necessary to use new technologies, new materials and methodologies for innovative executions. The need for higher quality at lower cost has driven a series of advancements in civil construction, as can be verified throughout history [1,2]. Studies on the energetic and environmental gain of the use of structural masonry in place of concrete structures estimate that there is a reduction of about 40% in energy expenditure and CO_2_ emissions [3].

Following such a trend, structural masonry, a constructive process characterized by using walls as the main supportive structure for the building, the walls have a double role as structural support and vertical sealing. Therefore, it can contribute to being a constructive model involving further constructive rationalization, structural design and more skilled labor [4,5,6,7,8].

The traditional technique for producing ceramic blocks is extrusion. In the present work, it was decided to replace the extrusion step by pressing the ceramic blocks in a methodology similar to that applied to soil-cement blocks, with subsequent firing to characterize the product. Therefore, a new ceramic product replacing the extrusion process by pressing red ceramic blocks such as the process performed with soil-cement bricks was developed [4,5,6,7,8]. The pressed blocks are burned later, enabling an improvement in the resistance and durability of those materials, which were denominated as pressed and burned blocks (PBB).

The structural masonry with dovetail blocks is an important construction method that generates less environmental impact, further rationalization and contributes to a higher sustainability in civil construction, once it reduces the production of wastes in a work, thus resulting in an improvement in the quality of life [9,10]. Structural blocks are characterized as to their technological properties, but they also must be assessed as to their performance in use. The best analysis is to trial the walls at their actual size. Nevertheless, the standards for the trials and dimensioning present alternatives to the wall samples in their actual size, which are the prism and small wall samples [1].

Prisms can be defined as the juxtaposition of two or more blocks connected by mortar joints destined for compression resistance trials. The definition of the amount of the prism layers must consider the influence that the height poses in order to attain the required resistance to compression. The height of the prism must be short enough for the trials not to be submitted to the slenderness effect. However, it is not too short to cause the restriction effect of the load application plates at the edges of the prism [11]. In this research, the three-layer prism analysis was chosen.

Prisms can present only horizontal joints or horizontal and vertical joints. Results from studies performed by some researchers suggest that the non-fulfillment of the vertical joints has little effect on the resistance to compression and in the deformation module of the masonry, being more relevant to the shear resistance [12,13,14,15,16,17,18,19,20,21,22,23,24,25,26,27,28,29]. As this is a study on the performance of the PBB facing simple compression, this article presents only cases where vertical mortar joints were not used.

The objective of this research was to present the results of research carried out with a new ceramic material, produced from the methodology proposed by the research group. Basic parameters for the execution of structural masonry projects were evaluated, thus verifying the compressive strength, the Poisson ratio and the deformation modulus of blocks, prisms and small walls of PBB. Although there are numerous researches on fired ceramic blocks, most of them are aimed at blocks produced by the extrusion technique. Thus, this work fills a gap in the literature on ceramic blocks produced by the pressing technique.

## 2. Materials and Methods

**Figure 1:**
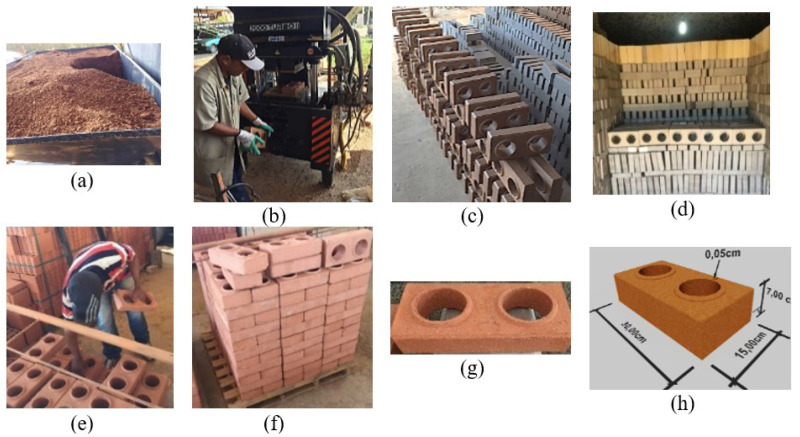
Manufacturing of the pressed and burned blocks: (**a**,**b**) manufacturing steps; (**c**) drying; (**d**) burn; (**e**,**f**) storage; (**g**,**h**) description of the block.

In this research it was used PBB measuring 30 × 15 × 7 cm^3^ produced by the authors at a brick factory in the Campos dos Goytacazes Potter Pole, according to a methodology adapted from Pedroti et al. [3], presenting several manufacturing steps for the production, drying, burn and availability of the blocks for the trials (Figure 1).

The PBB molding technique was based on the procedure used in the production of the blocks in soil cement, with adjustments to replace the use of cement in the firing process. The blocks were manufactured in press Eco Master 7000 Turbo II (Navegantes, Santa Catarina, Brazil) (Figure 1b) and fired at an average temperature of 900 °C, in Cerâmica Sardinha.

The blocks were studied as to their geometry, water absorption (WA), specific apparent mass (SAM), mechanical strength to compression ($f_{bk}$) and deformation module ($E_{b}$), according to the parameters set by the Brazilian standards [30,31,32,33,34].

**Figure 2:**
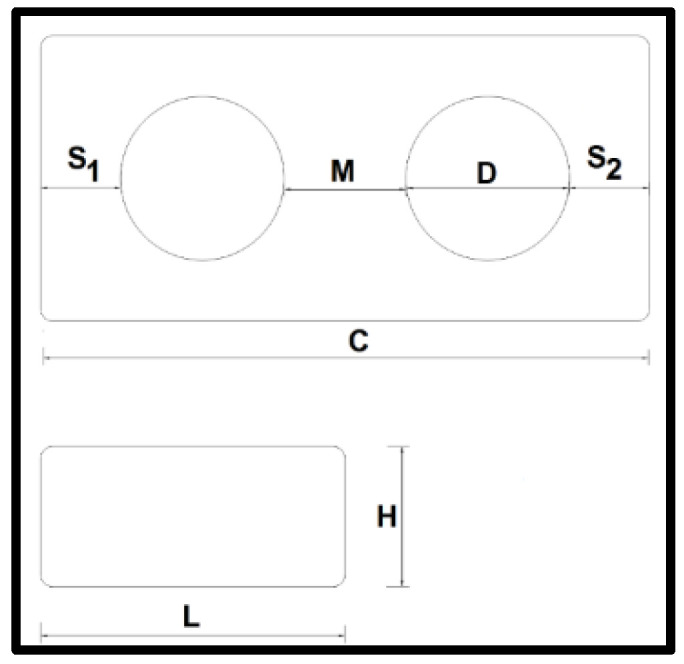
Scheme-indicated measure.

Figure 2 represents a schematic indicating the references used to determine the PBB measurements. Where L is width, H is height, C is length, S_1_ and S_2_ are the measures of the lateral septum in the larger direction, D is the diameter of the holes and M is the measure of the central part that is between the two holes of the PBB.

Thirty PBB samples were evaluated, and their dimensions compared with the 30 × 15 × 7 cm^3^ mold (length, width and height). Measurements were performed using a caliper with a precision of 0.05 mm.

The Water Absorption (WA) of the ceramic material is a useful parameter in determining the open porosity and knowing the rate is important to predict the durability of the ceramic piece, ensuring less water infiltration and greater durability and resistance to the natural environment to which the material is submitted. The tests were performed according to the Brazilian standard [34], applying the equation (Equation (1)) as follows:

(1) $$\mathrm{WA}\;=\left(\frac{m_{u}-m_{S}}{m_{S}}\right)\cdot 100\;\left(\%\right)\;$$

where: *m_u_* is the wet burnt mass; *m_S_* is the dry burnt mass of the specimen (in grams).

To obtain the water absorption rate, 13 samples of PBB were chosen for drying in an oven at a temperature of 110 °C for a period of 24 h, until the weight stabilized, defining the dry mass (*m_s_*). After cooling, the blocks were immersed in water at ambient temperature (23 °C) for a period of 24 h.

The apparent specific mass (ASM), another important property in the ceramic process that is related to the values of flexural strength of the pieces, water absorption and linear shrinkage, was determined by applying the equation (Equation (2)) as follows:

(2) $$\mathrm{ASM}\;=\frac{M}{V}\;$$

where: *M* is the mass of the ceramic piece; *V* is the volume of the fired or dried ceramic piece.

To determine the ASM, 13 samples of PBB were studied and their measurements and mass were determined to define the parameters *V* and *M*, respectively.

The characteristic strength ($f_{bk}$) is established through tests of individual compression strength ($f_{b}$), determined in relation to the gross area according to the Brazilian standard [34], from the equation (Equation (3)), defined in the standard [33].

(3) $$f_{bk,est}=2\cdot \left[\frac{f_{b\left(1\right)}+f_{b\left(2\right)}+f_{b\left(3\right)}+\cdots +f_{b\left(i-1\right)}}{i-1}\right]-f_{b\left(i\right)}$$

where:

$f_{bk,est}$: The estimated characteristic strength of the sample, in N/mm^2^;

$f_{b\left(1\right)};\;f_{b\left(2\right)};\;f_{b\left(3\right)};\cdots f_{b\left(i\right)}$: The values of individual compressive strength of the specimens of the sample, ordered increasing and the following:

$$i=\{\begin{array}{c} \frac{n}{2},\;if\;n\;even\;\\ \frac{n-1}{2},\;if\;n\;odd \end{array}$$

where: *n* is the number of blocks in the sample.

With the value of $f_{bk,est}$, $f_{bk}\;$ is determined as follows [33]:

$$\mathrm{If}\;f_{bk,est}\geq f_{bm},\;\mathrm{then}\;f_{bk}=f_{bm};$$

$$\;\;\;\;\mathrm{If}\;f_{bk,est}<\emptyset \cdot f_{b\left(1\right)},\;\mathrm{then}\;f_{bk}=\emptyset \cdot f_{b\left(1\right)};$$

$$\;\;\;\;\;\;\mathrm{If}\;\emptyset \cdot f_{b\left(1\right)}\leq f_{bk,est}\leq f_{bm},\;\mathrm{then}\;f_{bk}=f_{bk,est}.$$

where:

$f_{bm}$ is the average of the compression strengths of all specimens in the sample, in N/mm^2^;

$f_{b\left(1\right)}$ is the smallest resistance of all the specimens;

∅ is a tabled value.

The Brazilian standard [33] defines that at least 13 blocks be tested. In the present research, 15 PBB were tested to study the compressive strength. The estimated characteristic strength of the sample ($f_{ek}$), applicable for prisms ($f_{pk}$) and small walls ($f_{ppk}$), must be determined by Equation (4) as follows [33]:

(4) $$f_{ek,est}=2\cdot \left[\frac{f_{e\left(1\right)}+f_{e\left(2\right)}+f_{e\left(3\right)}+\cdots +f_{b\left(i-1\right)}}{i-1}\right]-f_{e\left(i\right)}$$

where:

$f_{bk,est}$: The estimated characteristic strength of the sample, in N/mm^2^;

$f_{b\left(1\right)};\;f_{b\left(2\right)};\;f_{b\left(3\right)};\cdots f_{b\left(i\right)}$: The values of individual compressive strength of the specimens of the sample, ordered increasing and the following:

$$i=\{\begin{array}{c} \frac{n}{2},\;if\;n\;even\;\\ \frac{n-1}{2},\;if\;n\;odd \end{array}$$

where: *n* is the number of elements in the sample.

With the value of $f_{bk,est}$, $f_{bk}\;$ is determined as follows [33]:

$$\;\;\mathrm{If}\;f_{ek,est}\geq 0.85f_{em},\;\mathrm{then}\;f_{ek}=0.85f_{em};$$

$$\mathrm{If}\;f_{ek,est}<\emptyset \cdot f_{e\left(1\right)},\;\mathrm{then}\;f_{ek}=\emptyset \cdot f_{e\left(1\right)};$$

$$\;\;\mathrm{If}\;\emptyset \cdot f_{e\left(1\right)}\leq f_{ek,est}\leq f_{bm},\;\mathrm{then}\;f_{ek}=f_{ek,est}.$$

where:

$f_{em}$ is the average of the compression strengths of all specimens in the sample, in N/mm^2^;

$f_{e\left(1\right)}$ is the smallest resistance of all the specimens;

∅ is a tabled value.

To study the behavior of the blocks in service, 6 prisms and 6 small walls were tested.

**Figure 3:**
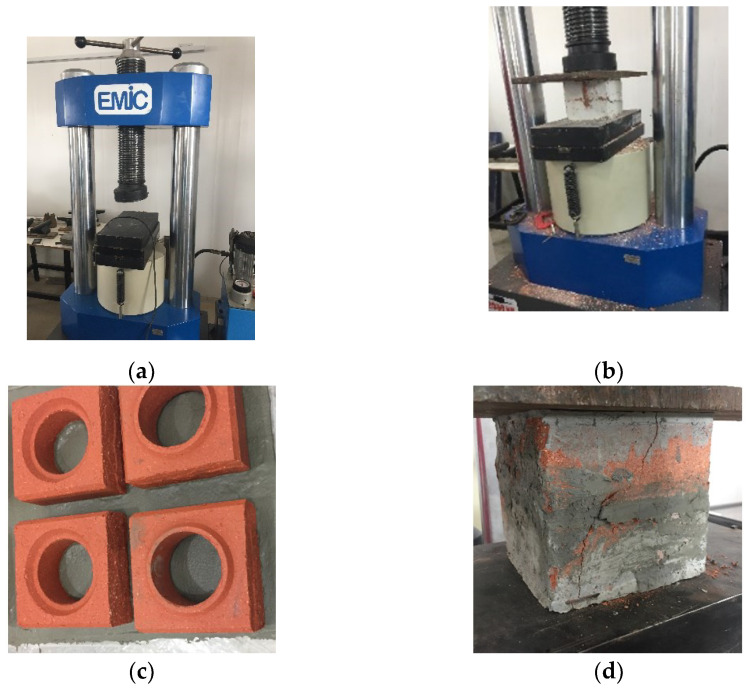
Experimental compressive tests: (**a**) 2000 kN EMIC Press; (**b**) PBB in the press; (**c**) capped PBB; (**d**) PBB capped in the press.

For the uniaxial simple compression trial, it was used a servo-hydraulic press (EMIC) from the Civil Engineering Laboratory at the State University Norte Fluminense Darcy Ribeiro (LECIV/UENF, Campos dos Goytacazes, Brazil) that works with load application speed regulation and data acquisition cell related to the strength applied along the time of 2000 kN load capacity (Figure 3a,b). The PBB were cut in half (such as soil-cement blocks) and capped with cement paste by using a 0.3 water/cement ratio to obtain the face regularization, as shown in Figure 3c,d. The surface capping aims to smooth the faces for the compression test, thus leaving them flat to ensure that the plates are parallel. This procedure also has the function of avoiding the accumulation of tensions in that place.

The compression test was carried out considering the load applied in the direction of the force acting in service, both for the blocks and for prisms and small walls, in the direction perpendicular to their length. The specimens were placed in the press so that the center of gravity coincided with the axis of the load of the plates of the press. The load application speed was controlled, and the press controls were regulated so that the applied tension progressively increased at a rate of 0.05 ± 0.01 (N/mm^2^)/s.

**Figure 4:**
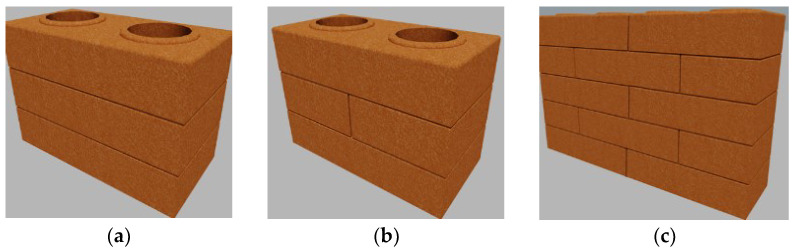
Prism models and small wall studied: (**a**) 3ISA Prism; (**b**) 3MSA Prism; (**c**) Small Wall (PPSA).

The mechanical strength to compression was directly set from the trial by the average (average compressive strength), the Weibull parameter (Weibull resistance) [35,36,37,38] and characteristic compressive strength, according to the methodology set by the Brazilian standard [33]. For the definition of structural masonry performance, it must be analyzed not only with the blocks but also with the interaction between them by the prism, small walls and wall studies. In this research, it was analyzed two three-layer prism models and a model of a small five-layer wall, without using fixing masonry (dry joints), as seen in Figure 4.

**Figure 5:**
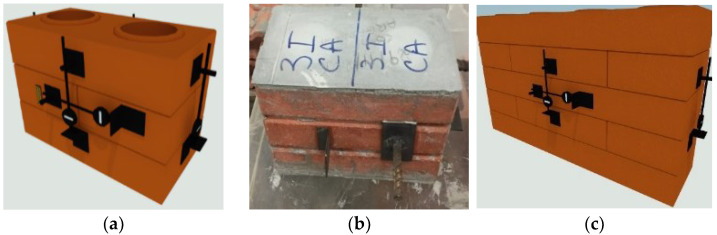
Prisms with indication of shafts for the LVDT: (**a**) prism models; (**b**) clad prism; (**c**) small wall model.

The determination of the mechanical compression strength of the prisms was carried out by the same press used for the block trial. For the data acquisition related to the displacements and deformations in the blocks and prisms, we used LVDT sensors glued to the pieces using adhesive LOCTITE 496 (Henkel, Jundiaí, Brazil) and reinforced with Epoxi Sikadur 32 adhesive glue, connected to a data acquisition system LYNX-type captured and interpreted by the AqDados and AqDanalysis 7.0 software (New York, NY, USA). This illustrates the positioning of the LVDTs in prisms and a small wall (Figure 5).

The deformation modulus was attained in relation to two secant curve points at 5% and 30% of the breakage load [31]. Equation (Equation (5)), according to the Brazilian standard [32] was used to determine the longitudinal deformation modulus, based on the stress–strain curve obtained from the compression test.

(5) $$E_{p}=\frac{\left(f_{p30}-f_{p5}\right)}{\left(\varepsilon_{30}-\varepsilon_{5}\right)}$$

where:

$E_{p}$: Deformation modulus;

$f_{p30}:$ Individual compressive strength, in relation to the gross area, referring to 30% of the breaking load, in N/mm^2^;

$f_{p5}:$ Individual compressive strength, in relation to the gross area, referring to 30% of the breaking load, in N/mm^2^;

$\varepsilon_{30}:$ Longitudinal deformation for 30% of the breaking load;

$\varepsilon_{5}:$ Longitudinal deformation for 5% of the breaking load.

The characteristic strength to simple compression (f_k_) of the ceramic masonry was taken from the characteristic resistance of the prisms, as it can be estimated to be 70% of the characteristic strength to simple compression of the prisms [31].

The results found by the mechanical trials for the PBB were statistically treated to allow extrapolations to be made through the verification in a specific limited amount of sampling. The statistical measurement parameters were set for the central position (average) and dispersion measurement (variance, standard deviation and coefficient of variance) were set.

The tests performed with ceramics present a certain discrepancy, so the samples need to have their results validated. In the present work, Chauvenet’s criterion was used to eliminate doubtful values and statistical validation of the results achieved. This is a method that is based on probability theory, considering the rational elimination of data outside the dominant trend. In this sense, the data are represented by the frequency function of the normal distribution, with maximum or minimum values being eliminated when doubts arise as to the variation of the measurements performed.

Furthermore, an analysis by the Weibull statistics was performed [38] to set the breakage parameters for the PBB. The likelihood of failure (breakage) was set by the probability estimator $P_{i}$, Equation (6), as follows:

(6) $$P_{i}=\frac{i-0.3}{N+0.4}\;$$

where:

i: The sample index;

N: The total number of samples measured––*n* = 14 (amount of non-rejected proof-bodies by the Chauvenet’s criterion).

## 3. Results and Discussion

The results obtained for the characterization of PBB, the object of several tests and analyses carried out with blocks, prisms and small walls, with due statistical treatment, are presented in this topic. The tests were carried out at the Civil Engineering Laboratory of the State University of Norte Fluminense Darcy Ribeiro.

The results were presented following the sequence described below: (i) Dimensional Characteristics of the Blocks; (ii) Technological Properties of Blocks; (iii) Compression Strength of Blocks (Average Strength and Characteristic Strength); (iv) Statistical Weibull Analysis; (v) Block Elasticity Parameters; (vi) Compression Strength of Prisms and Small Walls (Medium Strength and Characteristic Strength) and (vii) Efficiency Factor: Prisms and Small Walls.

It was sought in the geometrical analysis to check the PBB standardization as to the dimensions, deviation and flatness. Table 1 presents the average values attained from the statistical analysis at a 95% confidence level.

As can be observed in Table 1, the PBB complied with the maximum measurement variations both in the individual analysis and in the analysis of the average. Table 2 presents the results verified as to the technological properties of the PBB after the statistical treatment.

Results from the analysis of the water absorption rate (WA) indicate sufficient quality to employ the PBB as units for the sealing and structural masonry, as all the individual values and the average are within the range of limit for the standard, which is from 8 to 22% [4,5,6].

Pedroti et al. [4] worked with blocks manufactured both through manual and mechanical pressing, being burned at a lab at 900 °C, and it was found to have an average water absorption rate of 31.85% for manually pressed blocks and 30% for mechanically pressed blocks, values far above the maximum limit. Those values are 40% higher than the values attained in this study. This fact may be related to the higher compaction capacity of the press employed in this research (36 tons) and to the physical, chemical and mineralogical features of the clay mass employed to manufacture the blocks that presented a higher amount of melting materials (CaO e K_2_O).

Related to the SAM, the result points out an amount within the 1100–1400 kg/m^3^ range, the value forecasted for ceramic bricks and blocks. Table 3 below shows the values of the average strength to compression by considering the gross and net areas of the blocks after statistical treatment by Chauvenet’s criterion, by which sampling was rejected.

The average value of the strength to compression attained ($f_{bm}$) taken in relation to the gross area is higher than 3.0 N/mm^2^, the minimum value defined by the Brazilian standards to be used in structural masonry [33]. It was set at the characteristic strength to compression of the block ($f_{bk}$), which was 3.62 N/mm^2^, following what is defined by the Brazilian standard [33], also higher than the minimum for structural masonry. Compared to the amounts attained by other researchers who studied the PBB [4,5,6], the values found in this research presented better results as to the resistance to compression that may be associated with the pressing and the clay mass employed.

The mechanical strength of ceramics is quite influenced by the presence and form of defects in their microstructures, such as grains and pores. Therefore, one of the analyses that have been employed to assess the quality of the ceramic product has been the Weibull distribution, which considers the probability of failure over a specific tension since, mathematically, the Weibull parameter does not depend on the size of the sampling [38].

The Weibull module has been employed due to its flexibility of adequacy to different frequency distributions accumulated and to the process control involving failures in materials or parts along the time adequacy flexibility. The Weibull analysis correlates the mechanical strength of the material to its accumulated, such as the hood of breakage, an important criterion to set the confidence of the ceramic materials [35,36,37,38].

**Figure 6:**
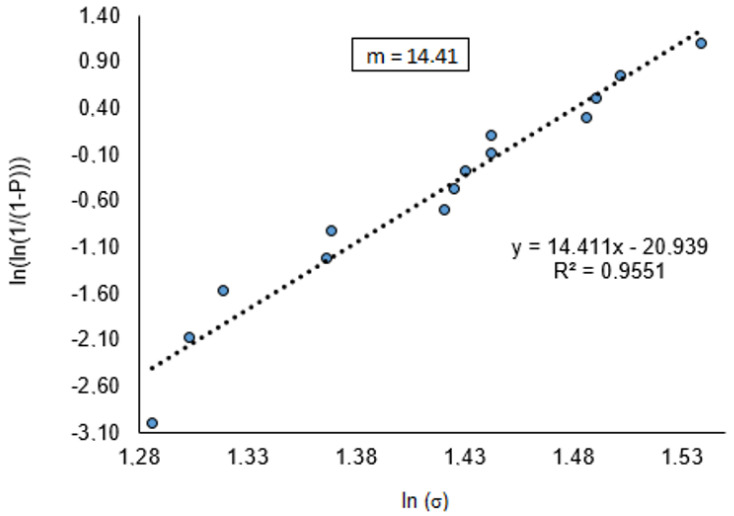
The Weibull chart (PBB).

Figure 6 presents the Weibull diagram (ln{ln [1/(1−P)]}) versus ln σ) for the PBB pieces. From the linear regression analysis of the experimental points, the Weibull parameter (m) was determined as follows, where: P is the probability estimator and σ is the failure stress for each sample tested.

As can be noted in Figure 6, the Weibull distribution is unimodal, with an R^2^ linearization of 0.955, indicating that the experimental data may be adjusted according to Weibull’s theory. The value of the Weibull parameter (m) is 14.41, which is within the acceptance range for ceramic materials (3 < m < 15), and the closer will be the upper limit, the lower will be the sampling dispersion [35,36,37,38]. 

**Figure 7:**
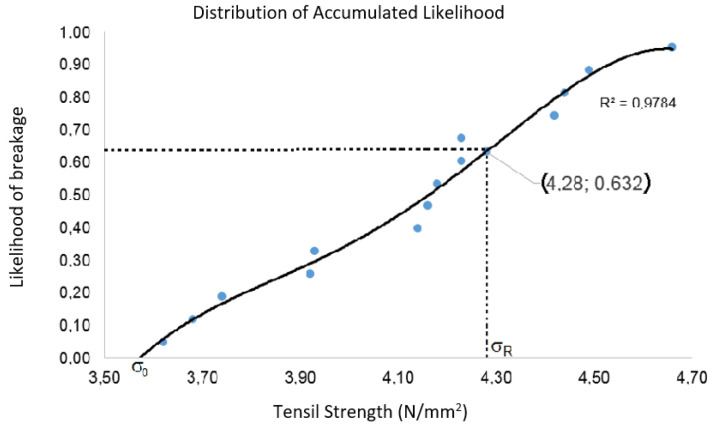
Distribution of the accumulated likelihood (PBB).

The characteristic Weibull tension ($\sigma_{R}$) was set, a parameter that indicates the likelihood of failure of the 63.2% block from the likelihood distribution of the breakage (Figure 7).

**Figure 8:**
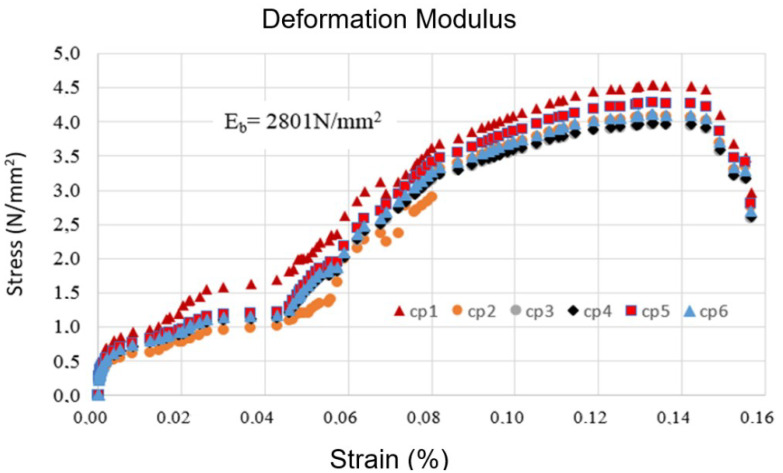
Strain curves-PBB.

The value set was 4.28 (N/mm^2^), as indicated in the graph in Figure 7, which is 3.6% higher than the average strength and 18.3% higher than the characteristic strength. The graph in Figure 8 presents the stress–strain curves attained for the PBB studied in this research.

As can be seen in the stress–strain curves of Figure 7, the behavior of the PBB is non-linear. Taking the deformation modulus of the tangent of the angle formed by the secant line to the graphs at the 5% and 30% points of the breakage stress [31], the average values set for the elasticity modules are presented in Table 4.

The values presented in Table 4 are very important to the structure design using PBB, and it was verified that the ratio between the characteristic compressive strength and the longitudinal elasticity module is as follows: $E_{b}=776f_{bk}$, which is 29.3% higher than the value recommended by the Brazilian standard for ceramic blocks [32]. In relation to the Poisson coefficient of 0.182, such an amount is 21.3% higher than the 0.15 set by the Brazilian standard [33] for masonry from the prism’s trials. 

**Figure 9:**
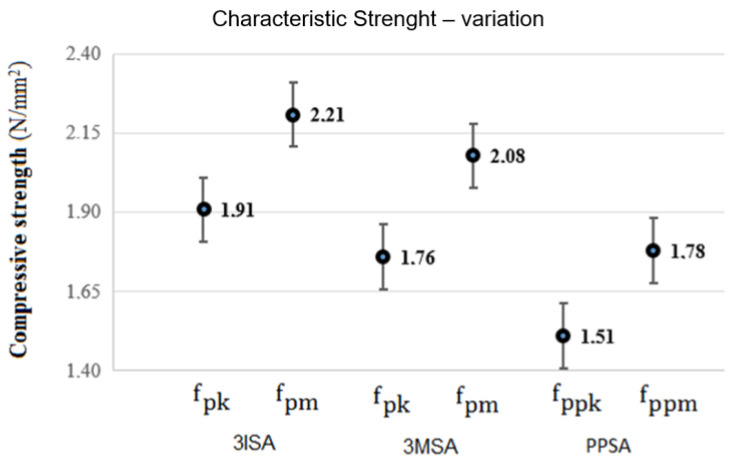
Compressive strength in N/mm^2^ (characteristic and average).

To define the performance of the PBB in use, 6 prisms of each model proposed were trialed and the results are presented in the charts in Figure 9. Results were assessed as to Chauvenet’s criterion, being approved at a 95% level of assurance [39].

The graph in Figure 9 presents the results obtained for the characteristic strength, indicating the variability around the value. In the graph shown in Figure 9, it can be noted that prisms in the 3ISA model present characteristic strength ($f_{pk})\;$8.5 % higher than 3MSA and 26.5% higher than the small walls (PPSA). As the mechanical resistance of the prisms does not present vertical joints but only complete overlapping blocks, it is the largest, but it represents in a less precise way the reality of a wall submitted to compression. Table 5 presents all the amounts attained to calculate the efficiency factor of the prisms/block, as well as the prism/prism ratio.

In ceramic materials, the forecasted prism/block efficiency ranges between 0.30 and 0.60, and the values found in this research are compatible with those parameters. Taking the tangent of the angle formed by the secant line to the graphs at the 5% and 30% points of the breakage stress as a deformation module, the longitudinal and transversal elasticity module are set for several prisms presented in Table 6 below, together with the amounts of the Poisson coefficient and the k value correlating the longitudinal deformation module (E) and the characteristic strength.

Upon the analysis of all values presented in Table 6, it can be noted that the ratio between the deformation module and the characteristic compressive strength in each case, represented by the letter k resulted in amounts between the reference value of the Brazilian standard [33], which is *k* = 600. Such a value complies with the minimum performance of the material.

It is also highlighted that in Table 6, the amounts attained are higher than 0.15, the reference value set by the Brazilian standard [33] for ceramic block prisms. The Brazilian standard [31], it is attained that the mechanical strength can be estimated at 1.3 N/mm^2^, enough for small-sized buildings.

The blocks, prisms and small walls presented fragile breakage, with failure by the traction in the blocks and the prisms and small walls, there was the formation of cracks at the sides and crushing of the central blocks.

## 4. Conclusions

The trials carried out had the main purpose to assess the physical and mechanical parameters of the PBB, which is still a little-studied product, aiming for its use in civil construction. In the assessment of the technological PBB parameters, it was verified that every normative parameter and those found in the market have been duly attended to. The blocks fired at a temperature of 890 °C presented dimensional averages, average deviations from the square and the flatness of the faces compatible with the requirements established by the Brazilian standard [33]. The water absorption rate of 20.84% is a value within the range indicated for masonry [33], but it needs to be controlled, as the variability of the result is still high, around 5%. The specific mass of 1210.82 kg/m^3^ of PBB is within the range for ceramics, not implying excessive loads on foundation structures, which further corroborates its use in structural masonry projects. PBB has also attended the minimum mechanical strength designated in the Brazilian standard, according to the block trials and it was found in amounts of 3.62, 4.10 and 4.23 N/mm^2^, respectively for the characteristic strength [33], average strength and the Weibull resistance. As the variability of measured values for the compressive strength was high, having even generated the rejection of value by Chauvenet’s criterion, it is concluded that it is more appropriate to use the characteristic strength value in structural calculations with PBB, since this value is moving so in favor of security.

The values of the deformation modules presented the forecasted results for ceramics. Both the prisms and the small wall presented a ratio between the longitudinal deformation module and the characteristic compressive strength higher than the reference value in the Brazilian standard, thus confirming the good ratio between the mechanical strength and deformability. As to the breakage mode, the PBB presented a fragile-type failure with a trend to failure in the block by the propagation of vertical cracks. It was observed that the first cracks occurred when the stress applied reached around 60% of the value of the last stress. The results indicate a good performance of the PBB in use with mechanical strength to compression of the masonry estimated in 1.3 N/mm^2^, thus being able to be employed in small works with simple architecture.

## Figures and Tables

**Table 1:** Geometrical characteristics.

Characteristics	Element	Allowed [11]	Verified
Individual Dimensional Tolerances (mm)	Width	±5	5.0
	Height		2.6
	Length		4.1
Average Dimensional Tolerances (mm)	Width	±3	3.0
	Height		2.1
	Length		2.9
Deviation in Relation to the Square (mm)	-	±3	1.2
Flatness of the Faces (mm)	-	±3	1.0

**Table 2:** Summary of the technological properties of the PBB.

Property	Average	Std. Deviation	Variation Coefficient (%)	Lower Limit	Higher Limit
WA (%)	20.84	0.94	4.53	20.32	21.36
SAM (kg/m^3^)	1210.82	25.98	2.15	1189.45	1232.20

**Table 3:** Mechanical strength to compression: average of the blocks.

	Gross Area	Net Area
Resistance (N/mm^2^)	4.13	5.28
Standard Deviation	0.32	
Coefficient of Variance (%)	7.68	

**Table 4:** Deformability modules of the blocks–gross area.

	$\mathrm{Longitudinal}\;(E_{b})$	$\mathrm{Transversal}\;\;\;(G_{b})$	Poisson Coefficient
Module (N/mm^2^)	2801	1185	0.182
Standard Deviation	0.10	0.02	-
Coefficient of Variance (%)	3.56	1.58	-

**Table 5:** Efficiency factor–prisms.

**Ratio**	**Efficiency Factor** **(*f_pk_*/*f_bk_*)**	**Efficiency Factor** **(*f_pm_/f_bm_*)**
3ISA	0.54	0.53
3MSA	0.50	0.51
PPSA	0.43	0.43

**Table 6:** Values of the deformation module-prisms.

Model	*f_k_* (N/mm^2^)	*E_p_* (N/mm^2^)	*G_p_* (N/mm^2^)	Poisson	$k=\frac{E}{f_{pk}}$
3ISA	1.91	1190	510	0.184	622.8
3MSA	1.76	1060	450	0.178	600.3
PPSA	1.51	980	420	0.172	649.4
